# Early clinical experience with eptinezumab: results of a retrospective observational study of patient response in the United States

**DOI:** 10.1186/s12883-023-03204-8

**Published:** 2023-04-20

**Authors:** Amaal J. Starling, Steven Kymes, Divya Asher, Seema Soni-Brahmbhatt, Meghana Karnik-Henry

**Affiliations:** 1 Mayo Clinic Arizona, 13400 East Shea Boulevard, Scottsdale, AZ 85259 USA; 2 Lundbeck LLC, Deerfield, IL USA

**Keywords:** Eptinezumab, Headache, Migraine, Monoclonal antibody, Preventive treatment

## Abstract

**Background:**

The efficacy and safety of eptinezumab for preventive migraine treatment in adults have been demonstrated in multiple, large-scale clinical trials. This non-interventional, retrospective, observational chart review was conducted to examine patient response to eptinezumab 100 mg or 300 mg every 12 weeks for 6 months in the clinical setting.

**Methods:**

Eight headache specialists who reported early clinical experience with eptinezumab enrolled the first adults (1–6 adults per clinician; age ≥ 18 years) who met predefined selection criteria (including ≥ 12-month history of migraine, ≥ 4 migraine days/month prior to eptinezumab initiation, receipt of ≥ 2 consecutive eptinezumab doses, and ≥ 12-week follow-up period), and provided detailed patient, disease, treatment, and outcome information via SurveyMonkey and standardized case-report forms.

**Results:**

Charts from 31 adults (median age, 49 years) with migraine (93.6% chronic) who received eptinezumab for the preventive treatment of migraine were reviewed. Most patients (26/31 [83.9%]) were initiated at 100 mg. Eptinezumab reduced mean headache frequency (24.3 monthly headache days [MHDs] at baseline; 17.1 MHDs at Month 6); mean migraine frequency (17.3 monthly migraine days [MMDs] at baseline; 9.1 MMDs at Month 6); attack severity (17/31 [54.8%] patients); acute headache medication use (12.5 acute medication days at baseline; 7.4 at Month 6); and patient-reported disability (11/22 [50.0%] severe at baseline; 7/19 [36.8%] at Month 6). More than three-quarters of patients (24/31 [77.4%]) perceived improved disability/function and most (30/31 [96.8%]) perceived eptinezumab to be well tolerated after 6 months. Most of the headache specialists reported that eptinezumab was well tolerated by patients (30/31 [96.8%]) and that the intravenous infusion experience was not challenging.

**Conclusions:**

Patients with migraine who received 6 months of preventive treatment with eptinezumab experienced reductions in migraine and headache frequency, disability, and acute medication use during the course of treatment.

**Supplementary Information:**

The online version contains supplementary material available at 10.1186/s12883-023-03204-8.

## Introduction

The release of calcitonin gene-related peptide (CGRP) may increase during acute migraine attacks, suggesting that it may be associated with migraine pathophysiology [1, 2]. Its role in initiating and perpetuating migraine is further supported by numerous studies demonstrating preventive benefits for those living with migraine following CGRP inhibition [1, 3, 4]. The humanized immunoglobulin G1 (IgG1) monoclonal antibody eptinezumab, which binds to CGRP with high affinity and blocks its binding to the CGRP receptor [5], has been approved in the United States, Canada, and European Union for the preventive treatment of migraine in adults [6–8].

The efficacy and safety of eptinezumab for the preventive treatment of migraine in adults have been demonstrated in multiple large-scale clinical trials, which revealed not only reductions in migraine and headache frequency, but also in acute migraine medication use and disability [9–14]. Evidence from post-marketing studies is needed to better understand eptinezumab efficacy and safety in the real world. The objective of this survey was to examine the patient response to eptinezumab 100 mg or 300 mg every 12 weeks for 6 months in the clinical setting. A secondary objective was to characterize headache specialists’ early experience with intravenous (IV) administration of eptinezumab.

## Methods

### Study design

This was a non-interventional, retrospective, observational chart review of patients of headache specialists who reported early clinical experience with eptinezumab from a Lundbeck-sponsored advisory board. The headache specialists are participating clinicians from academic, clinical, or consulting settings and were chosen to participate because they reported early clinical use of eptinezumab. There were four virtual advisory boards in September 2020 led by Dr. Amaal Starling and Dr. Richard Lipton in which 21 advisors participated. All advisory board attendees were invited to participate in the survey. “Early clinical experience” was defined as having treated a patient with eptinezumab during the first 6 months of its initial regulatory approval in the United States (approved April 2020); these clinicians were not chosen because they reported positive outcomes after initiating eptinezumab among their patients. Institutional review board approval was obtained for this protocol on 22 November 2021 (Advarra protocol number: 00059159).

### Patients

Each participating clinician enrolled the first adult patients (≥ 18 years of age) who met all predefined selection criteria. These criteria included a ≥ 12-month history of migraine with ≥ 4 migraine days per month prior to eptinezumab treatment; initiation of eptinezumab for the preventive treatment of migraine between April and October 2020; receipt of ≥ 2 consecutive doses of eptinezumab; and a minimum follow-up period of 12 weeks after the second eptinezumab dose. Patients using other preventive migraine treatments, oral medications, neuromodulatory devices, extracranial nerve blocks, and head and neck trigger-point injections were eligible for participation as long as these interventions had been stable for ≥ 3 months prior to initiating eptinezumab (≥ 6 months for onabotulinumtoxinA). Any concomitant hormonal therapy must also have been stable for ≥ 3 months prior to initiating eptinezumab.

Patients with uncontrolled and/or untreated psychiatric conditions (including those not controlled for a minimum of 6 months prior to initiating eptinezumab) were excluded from participation, as were those with a lifetime history of psychosis, mania, or dementia or who were pregnant, breastfeeding, or planning to become pregnant prior to initiating eptinezumab. Patients were also excluded if they had used any other CGRP monoclonal antibody within 1 month (or 3 months if dosed quarterly) prior to initiating eptinezumab; were actively participating in any other clinical drug trial; or had a condition that, in the opinion of the clinician, would make them unsuitable for the clinical study. The first 1‒6 fully completed patient surveys, in which patients met eligibility criteria, were submitted by participating clinicians and included in the survey. While the Lundbeck medical affairs team helped with initial study design, patient chart evaluation for eligibility criteria was completed under the guidance of Dr. Starling and did not involve Lundbeck. Lundbeck was not involved with the patient selection process.

### Data collection and handling

Participating headache specialists reviewed the charts of eligible patients and submitted requested data through SurveyMonkey using standardized case-report forms. All collected data were anonymized and transmitted over a hypertext transfer protocol secure (HTTPS) connection to the centralized data center, as maintained by SurveyMonkey, an online, web-based collection tool. Logins were protected via a transport layer security (TLS) protocol. Data at rest were encrypted using industry-standard encryption algorithms and strength. Data were analyzed at a group level as well as anonymized patient level. Due to incomplete responses or missing data points, data were only analyzed for the patient responses received. Therefore, in some instances (monthly migraine days, MMDs, pre-eptinezumab treatment, MMDs post-eptinezumab treatment, and acute medication use post-eptinezumab treatment) the sample size included in mean calculations was 28 instead of 31.

### Study assessments

The Early Physician Experience Survey (Supplement 1) included questions to assess baseline demographics (sex, race, ethnicity, and body mass index [BMI]) and comorbidities/risk factors (cardiovascular or vascular risk factors, neurological disorders, psychiatric disorders, non-migraine pain conditions, other conditions); disease history and characteristics prior to and after 6 months of eptinezumab treatment (migraine diagnosis, additional headache disorders, duration of illness, patient-identified most bothersome symptom [PI-MBS], presence/absence/type of aura, presence of daily headache, number of MMDs, and number of monthly headache days [MHDs]); changes in severity (decreased severity, no change in severity, increased severity, or no data available) and/or duration of individual migraine attacks after 6 months of treatment; the number and type of migraine preventive medications prior to eptinezumab initiation, at the time of the initial eptinezumab infusion, and 6 months after eptinezumab treatment; the number and type of acute migraine medications used in the 3 months prior to starting eptinezumab treatment, at the time of the initial eptinezumab infusion, and 6 months after eptinezumab treatment; details of eptinezumab dosing (initial dose, total doses over 6 months of treatment, and modification of treatment dose during initial 6 months); and disability as defined by the clinician prior to and 6 months after treatment (mild, moderate, severe, or N/A using the Migraine Disability Assessment [MIDAS] [15], 6-item Headache Impact Test [HIT-6] [16], or “Other” clinical disability scale). Both clinician-reported patient perspective and clinician perspective of treatment tolerability and impact on disability and function after 6 months of use were collected, including concerns about IV infusion experience, any change in comorbidities, clinician order for IV administration, and follow-up timing.

### Statistical analysis

Initially, 20 US clinicians were contacted for participation, and it was hypothesized that at least 10 clinicians would agree to participate and complete 3–4 chart reviews each, for a total of 30–40 chart reviews. There was an anticipated target of a minimum of 1‒2 patient case reports per clinician, with no more than 4 patients per clinician. However, if fewer clinicians participated, this patient target quantity per clinician could be increased.

All assessment data, including demographics, were summarized by subgroup (if applicable) using descriptive techniques. Summary statistics (mean, standard deviation, minimum and maximum values) were presented for continuous variables; counts and percentages were presented for categorical and binary variables. Due to incomplete reporting for several of the survey questions, sample sizes are provided with rates. MMDs, MHDs, and days of acute medication use were often reported by clinicians as a range; in these instances, the average of the range was reported.

## Results

### Patients

A total of 31 patient charts submitted by 8 headache specialists were eligible as determined by Dr. Starling and were included in analysis.

Each specialist enrolled, on average, 3.9 patients (range: 1‒6 patients/clinician) who had received at least 2 consecutive doses of eptinezumab for the preventive treatment of migraine. Patients had a median age of 49 years and were predominantly female (22/27 [81.5%]) and white (27/27 [100%]). Most patients had chronic migraine (CM; defined as > 14 MHDs; 29/31 [93.6%]) and more than one-half (18/31 [58.1%]) reported daily headache. A total of 6/31 (19.4%) patients had medication-overuse headache (MOH). Baseline demographic and clinical characteristics are summarized in Table 1.

**Table 1:** Baseline demographic and clinical characteristics

**Characteristic**	N*	
Age, median (range)	31	49.0 (23.0–72.0)
Sex, n/N (%)	27	
Male		5/27 (18.5)
Female		22/27 (81.5)
Race	27	
White, n/N (%)		27/27 (100)
Ethnicity	27	
Not Hispanic or Latino		27/27 (100)
BMI, median (range)	30	28.6 (16.0–47.3)
Cardiovascular risk factors, n/N (%)	30	
≥ 1 Cardiovascular risk factor		15/30 (50.0)
Hypertension		6/30 (20.0)
Obesity		5/30 (16.7)
High cholesterol		4/30 (13.3)
Stroke		1/30 (3.3)
Diabetes		1/30 (3.3)
Other		4/30 (13.3)
Concomitant conditions, n/N (%)		
Neurological disorder^a^	30	12/30 (40.0)
Psychiatric disorder^b^	29	19/29 (65.5)
Non-migraine pain condition	29	14/29 (48.3)
Migraine type	31	
Episodic		2/31 (6.5)
Chronic		29/31 (93.5)
Duration of illness	30	
< 1 year		0
1–5 years		4 (13.3)
5–10 years		5 (16.7)
10–15 years		4 (13.3)
> 15 years		17 (56.7)
Headache frequency (MHDs); mean (median; range)	31	24.3 (29.0; 8.0–30.0)
Migraine frequency (MMDs); mean (median; range)	28	17.6 (16.0; 8.0–30.0)
Disability, n (%) severe using MIDAS or HIT-6	22	11 (50.0)
Acute medication use, mean days/month (median; range)	31	13.5 (15.0; 0.0–30.0)

*BMI* Body mass index, *HIT-6* 6-item Headache Impact Test, *MHDs* Monthly headache days, *MIDAS* Migraine Disability Assessment, *MMDs* Monthly migraine days, *OSA* Obstructive sleep apnea, *PTSD* Post-traumatic stress disorder

^*^Number of participants reporting. ^a^Survey answer choices included epilepsy, multiple sclerosis, insomnia, OSA, and other. ^b^Survey answer choices included depression, anxiety, PTSD, and other

### Dosing

Most patients (26/31 [83.9%]) were initiated at a dose of 100 mg, which is the recommended starting dose according to the eptinezumab USPI; 5/31 (16.1%) were initiated at a dose of 300 mg. These 5 patients all had CM and reported, on average, 29.5 monthly headache days at baseline. A total of 9/31 (29.0%) patients had their dose modified (increased from 100 to 300 mg) during the initial 6 months of treatment. Reasons for dose modification were partial response to 100 mg (*n* = 3), wearing off/early washout (*n* = 2), response unsatisfactory to the patient (*n* = 1), patient willing to have dose increased (*n* = 1), and no reason provided (*n* = 2); 100% of these patients had CM and reported, on average, 25.0 monthly headache days at baseline.

### Improvement in headache/migraine frequency

**Fig.1:**
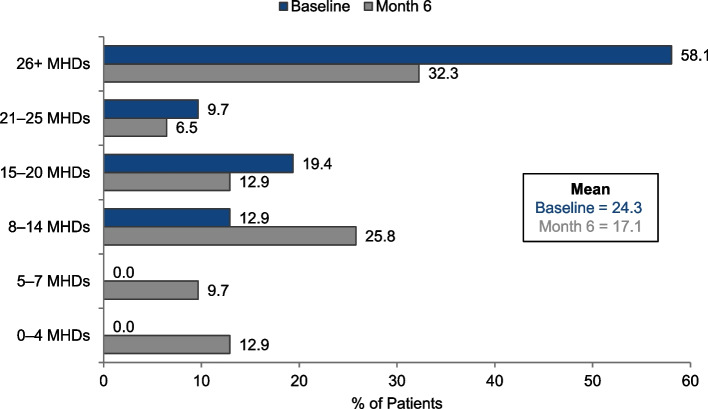
Monthly headache days (MHDs) at baseline and Month 6

**Fig. 2:**
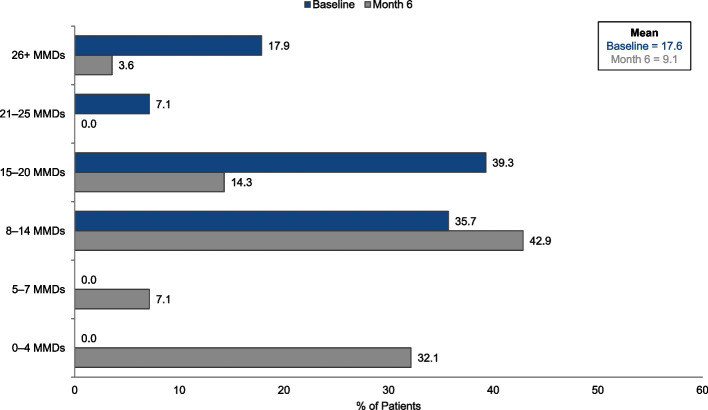
Monthly migraine days (MMDs) at baseline and Month 6

Six months of preventive treatment with eptinezumab reduced mean headache frequency (i.e., MHDs) from 24.3 days at baseline to 17.1 days at Month 6 (*n* = 31; Fig. 1) and the mean within-person change was 7.2 MHDs. Mean migraine frequency (i.e., MMDs) was reduced from 17.6 days at baseline to 9.1 days at Month 6 (Fig. 2) and the mean within-person change was 8.6 MMDs. Whereas most patients (29/31 [93.5%]) had CM (defined as > 14 MHDs) at baseline, only 8/31 (25.8%) met this criterion at Month 6. Six months of preventive treatment with eptinezumab reduced median headache frequency from 29.0 days at baseline (interquartile range [IQR] = 18.75–30) to 15.0 days (IQR = 7.75–30) at Month 6. Median migraine frequency was reduced from 16.0 days at baseline (IQR = 12–21) to 9.5 days (IQR = 3–12) at Month 6.

### Improvement in migraine attack severity

After 6 months of preventive treatment with eptinezumab, decreased severity of individual migraine attacks was reported in 17/31 (54.8%) patients, with no change reported in 11/31 (35.5%) and increased severity reported in 1/31 (3.2%).

### Improvement in disability

Relative to baseline, 6 months of preventive treatment with eptinezumab reduced patient-reported disability (50.0% [11/22] severe at baseline per MIDAS and/or HIT-6, and 66.7% [6/9] per “other disability scale used in clinical practice”, compared to 36.8% [7/19] reporting severe on MIDAS and/or HIT-6 and 50.0% [4/8] on “other disability” at Month 6). Per clinician report, more than three-quarters (24/31 [77.4%]) of patients perceived improved disability/function after 6 months of eptinezumab use, which aligned with the clinician perspective of patient improvement in disability/function (24/31 [77.4%]).

### Reduced acute headache medication use

**Fig. 3:**
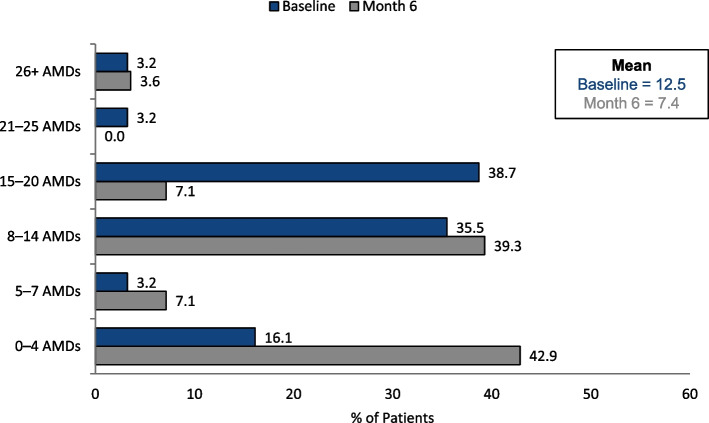
Acute medication days of use (AMDs) at baseline and Month 6

Eptinezumab treatment was associated with reduced acute medication days of use (12.5 mean acute medication days prior to starting treatment and 7.4 at Month 6 [*n* = 31]), as shown in Fig. 3. The median number of days of acute medication use was 12.5 days (IQR = 9.5–17.9) before treatment and 7 days (IQR = 1–12) at Month 6.

### Tolerability

Per clinician report, most patients (30/31 [96.8%]) perceived eptinezumab to be well tolerated after 6 months of use; clinicians also reported that for 96.8% (30/31) of patients, the IV infusion experience was not challenging.

## Discussion

In this chart review of 31 people living with migraine, cared for by 8 clinicians in a real-world setting, preventive treatment with eptinezumab was associated with reductions in headache and migraine frequency, attack severity, days of acute headache medication use, and functional disability. The changes in headache and migraine frequency observed over 6 months (–7.2 MHDs and –8.5 MMDs) are consistent with changes reported in patients who received eptinezumab in PROMISE-2, a randomized, placebo-controlled trial conducted in patients with CM (–8.2 and –8.8 MHDs over Weeks 1–12; –9.6 and –10.6 MHDs over Weeks 13–24; and –7.9 and –8.5 MMDs over Weeks 1–24 in the 100-mg and 300-mg groups, respectively) [12]. The changes in frequency observed in the current study shifted reclassification from a diagnosis of CM to one of episodic migraine (EM) in many patients; that is, 29/31 (93.6%) were considered to have CM at baseline, but only 8/31 (25.8%) did at Month 6.

Reductions in attack severity were reported in both the current real-world study (54.8% of patients demonstrated decreased attack severity) and in PROMISE-2 (proportion of severe attacks reduced 13.5% and 12.4% in the eptinezumab 100-mg and 300-mg groups, respectively vs 8.5% in the placebo group) [17].

Reductions in days of acute headache medication use were observed in the current survey and were consistent with reductions reported in PROMISE-2 (–5.1 days over Months 1–6 across both doses vs –3.3 and –3.5 days over Weeks 1–12 and –3.4 and –3.9 days over Weeks 13–24 with eptinezumab 100 mg and 300 mg, respectively) [12]. While the current study by design did not rely on electronic diary (eDiary) data, it probed lifetime estimates of preventive medication use, acute medication use months prior to starting eptinezumab treatment, and acute medication use while receiving eptinezumab treatment. In PROMISE-2, patients recorded their use of acute migraine medication in a daily eDiary over Weeks 1–24. In both studies, patients’ migraine medication use included acute and preventive medications, but the survey encompassed a wider range of headache medication use (such as gepant therapies).

The proportion of patients reporting severe disability (based on MIDAS and/or HIT-6 assessment) was reduced approximately 13% from baseline to Month 6 in the current study. This is a considerably smaller benefit than that reported in PROMISE-2, where there was an approximately 39% and 46% reduction from baseline in patients with severe disability (HIT-6) over Weeks 9–12 and Weeks 21–24, respectively, in the eptinezumab 100-mg group and a 47% and 49% reduction in the 300-mg group during the same time period [12]. It is worth noting that in the current study, 18/31 (58%) of survey responses collected from physicians indicated that their patients experienced daily headache, which may indicate greater overall disability at baseline. Further, due to limited sample size, HIT-6 and MIDAS scores were collated into one assessment of disability, which may also contribute to the lower values compared to PROMISE-2 data. This may also be attributable to limitations of the HIT-6, including the lack of time boundaries for 3 of the 6 questions of the measure in the clinical setting; however, additional research is needed to explore this discrepancy.

The majority of patients in this chart review had CM (93.6%) of long duration (56.7% for > 15 years), a population somewhat similar to that of PROMISE-2, which was conducted in patients with established CM (mean duration 11.8 years) [11]. However, patients in the current study were older (mean age 50.7 years vs 40.5 years in PROMISE-2), and many had conditions that would have made them ineligible for PROMISE-2 participation. For example, 15/30 (50%) survey patients had cardiovascular risk factors, including obesity (17%), hypertension (20%), high cholesterol (13%), stroke (3.3%), and diabetes (3.3%); PROMISE-2 excluded patients with any active, progressive, or unstable cardiovascular condition. Whereas CGRP is active within the cardiovascular system, cardiovascular risk associated with CGRP-inhibitor use appears to be low [18]. A recent pooled post hoc analysis of data from 4 eptinezumab clinical trials suggested that eptinezumab did not induce meaningful changes in measures of cardiovascular health (i.e., blood pressure, heart rate, concomitant cardiovascular medication use) and demonstrated a low, placebo-like incidence of cardiovascular adverse events [14]. While this study did not directly assess the effect of eptinezumab on patients with cardiovascular risk factors, patients with these risk factors still exhibited positive treatment outcomes, suggesting that treatment with eptinezumab may be efficacious in this patient population; however, further research is needed.

More than one-half (58.1%) of survey patients reported daily headache and 6/31 (19.4%) had MOH. Whereas benefits in patients with MOH have been described [19, 20], this is the first study to explore effects in patients with daily headache. Although the sample sizes here were limited, our findings suggest that the benefits of eptinezumab extend to even those patients with daily headache and those with MOH in the real-world setting.

A total of 9/31 (29.0%) patients in the current cohort had their dose increased from 100 to 300 mg during the initial 6 months of eptinezumab treatment. Indeed, 100% of these patients had CM and reported, on average, 23.5 monthly headache days at baseline.

### Study limitations

The results of this study must be interpreted in light of the fact that this was a small (*N* = 31), observational, retrospective chart review of patient response and clinical experience in patients who were among the first to be prescribed eptinezumab in the real-world setting and, therefore, may not be representative of all people who are candidates for preventive treatment with anti-CGRP medications. This study did not include primary data collection from patients; thus, more comprehensive details on real-world safety and tolerability of eptinezumab (such as adverse event reporting and changes in comorbidities) were not captured. Given this, conclusions on the safety and tolerability from this study should be interpreted with caution. In addition, migraine and headache days were self-reported to the clinician and not a headache diary; thus, findings are limited not only by recall bias, but also by what the clinician documented regarding the patient encounter, as all survey answers were derived directly from the patient chart. It is also important to consider that physicians invited to participate in the survey represent early eptinezumab adopters and may not be representative of neurologists in general in the US. Survey respondents were provided honoraria for their participation in the Lundbeck-sponsored advisory board but were not involved in the development of this manuscript. Survey respondents were not compensated for survey completion.

Patients who only had 1 infusion were not included in this chart review; thus, findings reflect patients who continued eptinezumab for ≥ 6 months. Those who continued treatment for 6 months were more likely to be experiencing greater benefits and fewer side effects, thus resulting in a selection bias.

## Conclusion

The results of this retrospective chart review, the first real-world evidence study to focus on eptinezumab, suggest that patients with migraine who received 6 months of preventive treatment with eptinezumab in the real-world clinical setting experienced reductions in migraine and headache frequency, disability, and acute medication use during the course of treatment, consistent with the phase 3 clinical trials.

## Supplementary Information

**Additional file 1.**

## Data Availability

In accordance with EFPIA’s and PhRMA’s “Principles for Responsible Clinical Trial Data Sharing” guidelines, Lundbeck is committed to responsible sharing of clinical trial data in a manner that is consistent with safeguarding the privacy of patients, respecting the integrity of national regulatory systems, and protecting the intellectual property of the sponsor. The protection of intellectual property ensures continued research and innovation in the pharmaceutical industry. Deidentified data are available to those whose request has been reviewed and approved through an application submitted to https://www.lundbeck.com/global/our-science/clinical-data-sharing.

## Abbreviations

BMI: Body mass index
CGRP: Calcitonin gene-related peptide
CM: Chronic migraine
HIT-6: 6-Item Headache Impact Test
HTTPS: Hypertext transfer protocol secure
IgG1: Immunoglobulin G1
IQR: Interquartile range
IV: Intravenous
MIDAS: Migraine Disability Assessment
MHDs: Monthly headache days
MOH: Medication-overuse headache
MMDs: Monthly migraine days
PI-MBS: Patient-identified most bothersome symptom
TLS: Transport layer security
