# Supplementary material for: Early clinical experience with eptinezumab: results of a retrospective observational study of patient response in the United States
Source: BMC Neurol. 2023 Apr 20;23:158. doi: 10.1186/s12883-023-03204-8 (PMC10116681; doi:10.1186/s12883-023-03204-8)
Supplement: Supplementary file 1 — Additional file 1. [file 12883_2023_3204_MOESM1_ESM.pdf]

Early Physician Experience Survey

This observational study, via retrospective chart review, aims to describe the early clinical experience of clinicians and patients following six months of eptinezumab treatment compared to baseline for the prevention of migraine.

First charts for patients treated with eptinezumab who meet the predefined inclusion/exclusion criteria may be selected.

Please utilize the following online form to enter the required medical information from each individual patient's chart. The Unique Patient Record Number is specific to the number assigned to your identified patient.

All information collected is anonymized, and will be evaluated at the group and individual patient level.

* Unique Patient Record Number:

* First Name (initial only):

Suggested question: ✓ ⊗

Early Physician Experience Survey

Baseline Demographics

Patient age at first eptinezumab dose:

[ ]

Gender identity:

- ○ Male
- ○ Female
- ○ Non-binary
- ○ Other (please specify)

[ ]

Race:

- ☐ American Indian/Alaska Native
- ☐ Asian
- ☐ Black/African American,
- ☐ Native Hawaiian/Pacific Islander
- ☐ White
- ☐ Other (please specify)

[ ]

Ethnicity:

- ○ Hispanic or Latino
- ○ Not Hispanic or Latino

Body mass index (BMI):

[ ]

Suggested question: ✓ ⊗

Early Physician Experience Survey

Comorbidities

Cardiovascular or Vascular Risk Factors (Select all that apply):

- [ ] MI
- [ ] Stroke
- [ ] Hypertension
- [ ] Diabetes
- [ ] Other (please specify)
- [ ] None of the above
- [ ] High cholesterol
- [ ] Obesity
- [ ] Information not available in the medical records

Neurological Disorders (Select all that apply):

- [ ] Epilepsy
- [ ] Multiple Sclerosis
- [ ] Insomnia
- [ ] Other (please specify)
- [ ] None of the above
- [ ] OSA
- [ ] Information not available in the medical records

Psychiatric (Select all that apply):

- [ ] Depression
- [ ] Anxiety
- [ ] PTSD
- [ ] Other (please specify)
- [ ] None of the above
- [ ] Information not available in the medical records

Nonmigraine pain conditions (Select all that apply):

- [ ] Fibromyalgia
- [ ] Temporomandibular Joint Pain
- [ ] Information not available in the medical records
- [ ] Other (please specify)

  ____

- [ ] None of the above

Other (Select all that apply):

- [ ] Allergic rhinitis
- [ ] Asthma
- [ ] SLE
- [ ] Other (please specify)

  ____

- [ ] None of the above

Suggested question: ✓ ⊗

Early Physician Experience Survey

Disease History Prior to Treatment

Migraine Diagnosis

(Note that the diagnosis of episodic migraine - for the purposes of this survey - is defined as 4 to 14 migraine headache days per month for the past three months)

(Select only one):

- ◯ Episodic Migraine
- ◯ Chronic Migraine
- ◯ Other (please specify)

  [ ]
- ◯ None of the above

Duration of Illness (Select only one):

How long has this patient had headaches with migraine features?

- ◯ < 1 year
- ◯ 1–5 years
- ◯ 5–10 years
- ◯ 10–15 years
- ◯ 15+ years
- ◯ Information not available in the medical records

Did the patient report suffering from aura?

- ◯ Yes
- ◯ No
- ◯ Information not available in the medical records

If the patient reported suffering from aura, please indicate what type:

	Visual	Sensory	Speech	Motor
Select all those that apply	☐	☐	☐	☐

Other (please specify)

[ ]

Did the patient report daily headache?

- Yes
- No

Did the patient have an additional / other headache disorder diagnoses?

- Yes
- No

What additional / other headache disorder diagnoses did the patient suffer from ?

	Menstrually-related Migraine (MRM)	Medication Overuse Headache (MOH)	Post-traumatic Headache (PTH)	Cervicogenic headache	New Persistent Daily Headache (NDPH)	Trigeminal Autonomic Cephalalgia (TAC)	Cluster Headache (CH)	Paroxysmal Hemicrania (PH)	Hemicrania Continua (HC)	Short-lasting, Unilateral, Neuralgiform headache attacks with Conjunctival injection and Tearing	Trigeminal Neuralgia (TN)
Select all those that apply	☐	☐	☐	☐	☐	☐	☐	☐	☐	☐	☐

Other (please specify)

Number of monthly migraine days prior to treatment (as defined in the clinical studies):

Number of monthly headache days prior to treatment (as defined in the clinical studies)?

What is the patient-identified most bothersome symptom associated with migraine prior to treatment? (If not identified in chart review, please put N/A)

Patient disability:

Please report the level of disability that the patient reported prior to receiving eptinezumab treatment

	Mild disability	Moderate disability	Severe disability	N/A
MIDAS	○	○	○	○
HIT-6	○	○	○	○
Other scale utilized in your practice	○	○	○	○

Other (please specify)

Please check the box if there is no data available for the rating scale above.

☐ No Data Available

Suggested question: ✓ ⊗

Early Physician Experience Survey

Treatment History

* Please enter the lifetime estimated number of distinct prescription preventive treatment options taken for migraine **prior to starting** eptinezumab:

Please indicate if the patient ever was prescribed any of the following medications **prior to treatment** with eptinezumab

AEDs:

- ☐ Divalproex/Sodium Valproate
- ☐ Topiramate
- ☐ Carbamazepine
- ☐ Other AED

Antihypertensives:

- ☐ Metoprolol
- ☐ Propranolol
- ☐ Timolol
- ☐ Atenolol
- ☐ Candesartan
- ☐ Lisinopril
- ☐ Clonidine
- ☐ Guanfacine
- ☐ Nebivolol
- ☐ Pindolol
- ☐ Other antihypertensive

Antidepressant:

- ☐ Amitriptyline
- ☐ Venlafaxine
- ☐ Other antidepressant

Neurotoxin:

- [ ] OnabotulinumToxinA
- [ ] Other neurotoxin

Neuromodulatory device:

- [ ] Neuromodulatory device

Anti-CGRP Therapy

- [ ] Anti-CGRP Monoclonal Antibody
- [ ] GEPANT

Other:

Suggested question: ✓ ⊗

Early Physician Experience Survey

Treatment History

* Please enter the estimated number of distinct prescription preventive treatment options taken **at the time of the initial** eptinezumab infusion:

Please indicate if the patient was taking any of the following medications **at the time of the initial** eptinezumab infusion

AEDs (Select all that apply):

- ☐ Divalproex/Sodium Valproate
- ☐ Topiramate
- ☐ Carbamazepine
- ☐ Other AED

Antihypertensives (Select all that apply):

- ☐ Metoprolol
- ☐ Propranolol
- ☐ Timolol
- ☐ Atenolol
- ☐ Candesartan
- ☐ Lisinopril
- ☐ Clonidine
- ☐ Guanfacine
- ☐ Nebivolol
- ☐ Pindolol
- ☐ Other antihypertensive

Antidepressant (Select all that apply):

- ☐ Amitriptyline
- ☐ Venlafaxine
- ☐ Other antidepressant

Neurotoxin (Select all that apply):

- [ ] OnabotulinumToxinA
- [ ] Other neurotoxin

Neuromodulatory device:

- [ ] Neuromodulatory device

Anti-CGRP Therapy

- [ ] Anti-CGRP Monoclonal Antibody
- [ ] GEPANT

Other:

____

Suggested question: ✓ ⊗

Early Physician Experience Survey

Treatment History

* Please indicate the total number of days per month of acute medication use in the **last 3 months prior to starting** eptinezumab (list migraine days per month):

Please select any acute medications that the patient was prescribed or recommended **in the last 3 months prior to** starting eptinezumab

NSAIDS (Select all that apply):

- ☐ Acetylsalicylic acid
- ☐ Acetaminophen
- ☐ Ibuprofen
- ☐ Naproxen
- ☐ Other NSAID
- ☐ Other OTC simple or combination analgesic

Triptans (Select all that apply):

- ☐ Sumatriptan (Imitrex)
- ☐ Rizatriptan (Maxalt)
- ☐ Naratriptan (Amerge)
- ☐ Zolmitriptan (Zomig)
- ☐ Eletriptan (Relpax),
- ☐ Almotriptan (Axert)
- ☐ Frovatriptan (Frova)

Gepants (Select all that apply):

- ☐ Ubrogepant
- ☐ Rimegepant

Ditans (Select all that apply):

- ☐ Lasmiditan

Opioids:

- [ ] Opioid
- [ ] Other

Prescription combination analgesic:

- [ ] Fioricet
- [ ] Other (please specify)

Neuromodulatory device:

- [ ] Neuromodulatory device

Suggested question: ✓ ⊗

Early Physician Experience Survey

Treatment History

* Please indicate the lifetime estimated number of distinct acute treatment options taken for migraine **prior to starting** eptinezumab:

[ ]

Please indicate any acute medication that the patient was prescribed or recommended in their entire lifetime **prior to starting** eptinezumab

NSAIDS (Select all that apply):

- [ ] Acetylsalicylic acid
- [ ] Acetaminophen
- [ ] Ibuprofen
- [ ] Naproxen
- [ ] Other NSAID
- [ ] Other OTC simple or combination analgesic

Triptans (Select all that apply):

- [ ] Sumatriptan (Imitrex)
- [ ] Rizatriptan (Maxalt)
- [ ] Naratriptan (Amerge)
- [ ] Zolmitriptan (Zomig)
- [ ] Eletriptan (Relpax),
- [ ] Almotriptan (Axert)
- [ ] Frovatriptan (Frova)

Gepants (Select all that apply):

- [ ] Ubrogepant
- [ ] Rimegepant

Ditans (Select all that apply):

- [ ] Lasmiditan

Opioids:

- [ ] Opioid
- [ ] Other

Prescription combination analgesic:

- [ ] Fioricet
- [ ] Other (please specify)

Neuromodulatory device:

- [ ] Neuromodulatory device

* What non-migraine medications was the patient on at the time of the first infusion?

Suggested question: ✓ ⊗

Early Physician Experience Survey

Eptinezumab Dosing

Initial dose of eptinezumab

- ◯ 100mg
- ◯ 300mg

Other (please specify)

Has the patient received at least 2 infusions of eptinezumab during the past 6 months

- ☐ Yes
- ☐ No
- ☐ Other (please specify)

How many total doses of eptinezumab were given over the 6 months of treatment?

Number of 100mg:

Number of 300mg:

Was dosing of eptinezumab modified during the initial 6 months of treatment?

- ◯ Yes
- ◯ No

If yes, how and why was it modified?

Suggested question: ✓ ⊗

Early Physician Experience Survey

Post-eptinezumab Administration Disease Characterization

* Please enter the number of monthly **headache** days (as defined in the clinical studies) after 6 months of eptinezumab use (#/ per month):

[ ]

* Please enter the number of monthly **migraine** days (as defined in the clinical studies) after 6 months of eptinezumab use (#/ per month):

[ ]

Please enter any change in severity of individual migraine attacks (Select only one):

- ○ Decreased severity of individual migraine attacks
- ○ No change in the severity of individual migraine attacks
- ○ Increased severity of individual migraine attacks
- ○ No Data Available

Please enter the change in duration of individual migraine attacks (Select only one):

- ○ Shorter duration of individual migraine attacks
- ○ No change in the duration of individual migraine attacks
- ○ Longer duration of individual migraine attacks
- ○ No Data Available

Please enter the change in patient-identified most bothersome symptom associated with migraine after 6 months of eptinezumab use (Select only one)

- ○ Improvement in the patient-identified most bothersome symptom
- ○ No impact on the patient-identified most bothersome symptom
- ○ Worsening of the patient-identified most bothersome symptom
- ○ No Data Available

Please enter which symptom - that was associated with the patient-identified most bothersome symptom - changed after 6 months of eptinezumab use:

[ ]

Please enter the disability after 6 months of treatment with eptinezumab:

	Mild disability	Moderate disability	Severe disability	N/A
MIDAS	◯	◯	◯	◯
HIT-6	◯	◯	◯	◯
Other	◯	◯	◯	◯

Other (please specify)

Please check the box if there is no data available for the rating scale above.

☐ No Data Available

Suggested question: ✓ ⊗

Early Physician Experience Survey

Post-eptinezumab Administration Acute Medication Use

*

Please enter the number of days per month of acute treatment use in the last 3 months **while on** eptinezumab treatment (average number of acute medication days per month):

Please indicate any acute medications that were prescribed or recommended in the past 3 months **while on** treatment with eptinezumab

NSAIDS (Select all that apply):

- [ ] Acetylsalicylic acid
- [ ] Acetaminophen
- [ ] Ibuprofen
- [ ] Naproxen
- [ ] Other NSAID
- [ ] Other OTC simple or combination analgesic

Triptans (Select all that apply):

- [ ] Sumatriptan (Imitrex)
- [ ] Rizatriptan (Maxalt)
- [ ] Naratriptan (Amerge)
- [ ] Zolmitriptan (Zomig)
- [ ] Eletriptan (Relpax),
- [ ] Almotriptan (Axert)
- [ ] Frovatriptan (Frova)

Gepants (Select all that apply):

- [ ] Ubrogepant
- [ ] Rimegepant

Ditans (Select all that apply):

- [ ] Lasmiditan

Opioids:

- [ ] Opioid
- [ ] Other

Prescription combination analgesic:

- [ ] Fioricet
- [ ] Other (please specify)

Neuromodulatory device

- [ ] Neuromodulatory device

Suggested question: ✓ ⊗

Early Physician Experience Survey

Post-eptinezumab Administration Acute Medication Use

Were there any changes in prescription preventive treatment options (in addition to or apart from any changes in eptinezumab use) after 6 months of eptinezumab use?

- ○ Yes
- ○ No

If yes, what were the changes (select all that apply)?

- ☐ Stopped oral treatment options due to improvement
- ☐ Stopped oral treatment options due to tolerability
- ☐ Added oral treatment options due to inadequate benefit
- ☐ Added non-oral treatment options due to inadequate benefit
- ☐ Other (please specify)

Suggested question: ✓ ⊗

Early Physician Experience Survey

Post-eptinezumab Administration Acute Medication Use

Please indicate the current prescription preventive treatment options, not including eptinezumab, **after 6 months of** eptinezumab use (Select all that apply):

[ ]

Please indicate any preventive therapies that were prescribed or recommended **after 6 months of** eptinezumab treatment

AEDs (Select all that apply):

- ☐ Divalproex/sodium
- ☐ Valproate
- ☐ Topiramate
- ☐ Carbamazepine
- ☐ Other AED

Anti-hypertensives (Select all that apply):

- ☐ Metoprolol
- ☐ Propranolol
- ☐ Timolol
- ☐ Atenolol
- ☐ Candesartan
- ☐ Lisinopril
- ☐ Clonidine
- ☐ Guanfacine
- ☐ Nebivolol
- ☐ Pindolol
- ☐ Other antihypertensive

Anti-depressant (Select all that apply):

- ☐ Amitriptyline
- ☐ Venlafaxine
- ☐ Other antidepressant

Neurotoxin (Select all that apply):

- [ ] OnabotulinumToxinA
- [ ] Other neurotoxin

Neuromodulatory device

- [ ] Neuromodulatory device

Anti-CGRP Therapy

- [ ] Anti-CGRP monoclonal antibody
- [ ] GEPANT

Other:

Suggested question: ✓ ⊗

Early Physician Experience Survey

Patient Perspective

Patient perspective of eptinezumab’s tolerability after 6 months of use (Select only one):

- ◯ Well tolerated
- ◯ Not well tolerated

Patient perspective of eptinezumab’s impact on disability and function after 6 months of use (Select only one):

- ◯ Improvement
- ◯ No impact
- ◯ Worsening

Patient concerns about receiving an infusion for migraine prevention:

- ◯ Yes
- ◯ No

If yes, please indicate concern:

Any change in comorbidities:

- ◯ Yes
- ◯ No

If yes, please indicate change:

Was there any change in non-migraine medications after 6 months of eptinezumab use?

- ◯ Yes
- ◯ No

If yes, please indicate change:

Suggested question: ✓ ⊗

Early Physician Experience Survey

Clinician Perspective

Clinician perspective of the patient's eptinezumab tolerability after 6 months of use (Select only one):

- ◯ Well tolerated
- ◯ Not well tolerated

Clinician perspective of the patient's eptinezumab impact on disability and function after 6 months of use (Select only one):

- ◯ Improvement
- ◯ No impact
- ◯ Worsening

Clinician perspective about IV infusion experience for this specific patient:

- ◯ Not challenging
- ◯ Challenging

If challenging, why (e.g. access to infusion center, writing the order, support staff training, side effects, cost, prior authorization, etc)?

[ ]

How are you writing the order for IV administration for eptinezumab for this specific patient?

- ◯ 1x Order
- ◯ Standing order of every 12 weeks for 6 months
- ◯ Standing order of every 12 weeks x 1 year
- ◯ Other (please specify)

[ ]

Do you require the patient to come in for follow-up prior to providing new order for repeat infusion?

- ◯ Yes
- ◯ No

Was your choice based on:

- ◯ Clinical preference
- ◯ Dictated by insurance
- ◯ Other (please specify)

How often are you seeing this specific patient on IV eptinezumab for follow-up? (Select all that apply)

- ☐ 1 month
- ☐ 3 months
- ☐ 6 months
- ☐ Other (please specify)

How often are you seeing this specific patient on IV eptinezumab for follow-up? (Select all that apply)

- ☐ 1 month
- ☐ 3 months
- ☐ 6 months
- ☐ Other (please specify)

Suggested question: ✓ ⊗
